# Impact on gait biomechanics of using an active variable impedance prosthetic knee

**DOI:** 10.1186/s12984-016-0159-0

**Published:** 2016-06-10

**Authors:** Matthew R. Williams, Susan D’Andrea, Hugh M. Herr

**Affiliations:** Louis Stokes Cleveland VA Medical Center, Cleveland, OH USA; Department of Biomedical Engineering, Case Western Reserve University, Cleveland, OH USA; Providence VA Medical Center, Providence, RI USA; Department of Orthopedics, Brown University, Providence, RI USA; Media Lab, Massachusetts Institute of Technology, Cambridge, MA USA

## Abstract

**Background:**

An above knee amputation can have a significant impact on gait, with substantial deviations in inter-leg symmetry, step length, hip exertion and upper body involvement even when using a current clinical standard of care prosthesis. These differences can produce gait that is less efficient and less comfortable, resulting in slower and shorter distance walking, particularly with long term use.

**Methods:**

A robotic variable impedance prosthetic knee (VI Knee) was tested with five individuals (*N* = 5) with unilateral amputation above the knee at fixed speeds both above and below their normal walking speed. Subject gait was measured as they walked along an instrumented walkway via optical motion capture and force plates in the floor. Each subject’s gait while using the VI Knee was compared to that while using their standard of care knee (OttoBock C-Leg).

**Results:**

Significant differences (p < 0.05) in walking between the standard of care and variable impedance devices were seen in step length and hip range of motion symmetries, hip extension moment, knee power and torso lean angle. While using the VI Knee, several subjects demonstrated statistically significant improvements in gait, particularly in increased hip range of motion symmetry between affected and intact sides, greater prosthesis knee power and in reducing upper body involvement in the walking task by decreasing forward and affected side lean and reducing the pelvis-torso twist coupling. These changes to torso posture during gait also resulted in increased terminal stance hip flexion moment across subjects. Detriments to gait were also observed in that some subjects exhibited decreased step length symmetry while using the VI Knee compared to the C-Leg.

**Conclusions:**

The knee tested represents the potential to improve gait biomechanics and reduce upper body involvement in persons with above knee amputation compared to current standard of care devices. While using the VI Knee, subjects demonstrated statistically significant improvements in several aspects of gait though some were worsened while using the device. It is possible that these negative effects may be mitigated through longer term training and experience with the VI Knee. Given the demonstrated benefits and the potential to reduce or eliminate detriments through training, using a powered device like the VI Knee, particularly over an extended period of time, may help to improve walking performance and comfort.

## Background

More than 270,000 persons with amputation (PWA) above the knee currently reside in the United States, with an amputation incidence rate of 39,000 new cases each year [1]. In the Veteran community, the Veteran’s Health Administration performs about 1500 to 2000 above-knee amputations each year with vascular disease as a result of diabetes being the most prevalent reason for surgery [2]. In terms of service-related injuries, the military actions over the past 14 years have seen many service personnel who have suffered trauma requiring above knee amputation. It is estimated that 34.5 % of individuals with combat injuries needing amputation required at least one above knee amputation [3].

Current standard of care prostheses result in gait that displays marked asymmetry including differences in step length, hip moment and torso involvement [4–7]. This results in changes in gait symmetry (step length and hip range of motion), an indicator of altered joint loading that can have a deleterious impact on the intact side limb, possibly leading to a higher prevalence of back and hip pain and knee osteoarthritis [8, 9]. Today’s advanced prosthetic knees (such as the Otto Bock C-Leg and Genium and the Ossur Rheo Knee) have been shown to reduce both hip moment and the metabolic requirements of using such a device, especially at speeds faster and slower than customary walking speed [10, 11], decreasing the overall exertion and effort felt by the user. The newer generations of prostheses have also shown some improvement in step length symmetry under controlled speeds [12] but no studies have shown them to improve upper body motion over simpler prostheses [7]. Given the relatively young age at which current combat Veterans are being fitted with knee prostheses and their expected life expectancy and long term use of such devices, the need for a prosthesis which improves gait symmetry and reduces abnormal joint loading is significant. Additionally, more elderly Veterans who are recent PWAs due to vascular disease may benefit from a prosthesis which requires less effort to use which may result in greater ambulation and mobility.

Research into powered prosthetic knees has been conducted for several decades [13–17], however, a viable, self-contained device with all actuators, power and computation/control on board has been demonstrated only recently. Goldfarb has developed a powered knee-ankle prosthesis that can reproduce some of the kinematics of able-bodied gait over level ground [18] as well as walking up an incline [19]. In terms of commercial devices, Ossur has developed the Power Knee, a position controlled, direct transmission knee that uses echo control of the intact leg to control prosthetic knee motion [20, 21]. These previous approaches to restore knee function have not ideally taken advantage of mechanisms which replicate the passive properties of the leg during ambulation or the potential benefits of energy storage and return as seen in the organic leg. One of the main disadvantages of such systems is the substantial power consumption required to replicate natural gait with a direct-drive transmission. This results in lower battery life or requires a larger battery to meet daily step counts. By not leveraging the ability to store and release mechanical energy via passive elastic elements in the prosthesis, such devices ignore a key opportunity to optimize the size and weight of powered knee systems [22].

**Fig. 1:**
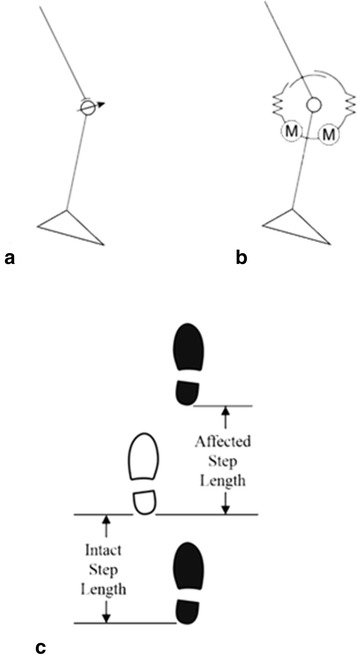
Schematic representation of the mechanics of the C-Leg (**a**) with its variable damper and VI-Knee (**b**) with its series elastic actuators in an agonist-antagonist arrangement for flexion and extension. An illustration of how step length is defined for this work with the shaded footprint representing the prosthetic foot and the open print the intact foot (**c**)

In contrast to the powered prosthetic knee approaches discussed above, during able-bodied level ground walking, energy is primarily conserved by transfer, storage and later release of potential energy with little input power needed from propulsive forces from the hip. This energy is stored in the elastic tendons of the legs or by lifting the body’s center of mass [23]. Biomechanical models of gait, using passive actuators (such as springs) along with clutches to control the activation of these actuators, have demonstrated that a large portion of the leg’s motion can be achieved using passive elastic elements [24]. During level ground walking the bulk of the muscle activity at the knee is related to energy absorption and storage than actively generating power [25]. Current prosthetic knees (such as the OttoBock C-Leg studied in this work) dissipate mechanical energy, only providing braking torque on the knee during swing phase (Fig. 1a) [26]. Different from this passive approach, series elastic actuators can be used in prostheses to provide net positive energy with low electrical power by taking advantage of energy storage in the elastic elements during gait, as well as accurately replicate the general joint kinematics and kinetics of the knee. In addition to their capability for energy storage, this type of actuator allows for more biomimetic motion and has inherent impact tolerance due to their elastic components (critical for a walking device) and have been used in a number of walking robots and robotic prostheses [27, 28].

The work of Martinez-Villalpando and Herr has developed a variable impedance knee prosthesis (VI Knee) that uses an agonist-antagonist arrangement of two series elastic actuators to control knee position and impedance in addition to providing propulsive power in gait (Fig. 1b) [22]. This opposed actuation method enables each actuator to operate often unloaded, chiefly to control joint stiffness. This results in a significant reduction in the electrical demands of the device. By using series elastic actuators, the device can not only store and release mechanical energy (via the elastic elements of the actuators) during walking, but can also help to lift the center of mass by producing net positive power in late stance. Variable impedance control adjusts the knee output torque (either braking or producing positive torque) as a function of its rotational velocity, thereby producing a more biomimetic gait. In early studies, individuals with above knee amputations walking on level ground at their normal, customary walking speed were observed to have a 6.8 % reduction in their metabolic effort due to using this device compared to their conventional prosthesis [29].

This study investigated the biomechanical differences between using the VI Knee compared to a C-Leg (Otto Bock, Duderstadt, Germany), the current standard of care device for active walkers. It was hypothesized that by using a knee with active flexion and extension, the user’s gait would be improved at speeds both above and below their customary walking speed due to increased joint symmetry as well as reduced involvement of the affected side hip in controlling the motion of the lower leg.

## Methods

The device tested in this study was the VI Knee [29], a powered prosthetic knee developed at The Massachusetts Institute of Technology (MIT). Eleven unilateral, PWAs above the knee were recruited through connections with local prosthetists based on the following inclusion criteria: Height over 1.5 m, weight between 83 and 113 kg, age between 20 and 70 years, and rated as a level K3 or above ambulator (defined as being able to voluntarily vary walking speed and walk without hand-held assistive devices), and be experienced (greater than 1 year) C-Leg users. Weight and height criteria were set by the weight and length of the VI Knee so that it approximately matched the weight and length of the missing lower leg. Recruited subjects covered a breadth of years since amputation and represented a span of residual limb lengths (defined as the proportion (listed as a percentage in this work) of intact femur length remaining after amputation). Of those recruited, five subjects completed the experiments with the other six dropping out shortly after screening due to increased time demands at work (*n* = 3), injuries at home not associated with the study (*n* = 2) and one exceptionally tall and heavy participant who despite meeting the inclusion criteria was unable to properly walk with the VI Knee due to over-torqueing the device when turning corners. Subject demographics of the participants who completed the study can be seen in Table 1. All subjects gave informed consent to participate and experimental protocols were approved by the Providence VA Medical Center (PVAMC) IRB.

**Table 1:** Subject Demographics

Subject	Cause of amputation	Experience (yrs)	Residual limb length	Prosthetic foot
S2	Trauma	27	90 %	Ossur Low Profile Vari-Flex
S3	Cancer	41	100 %	College Park Trustep
S4	Trauma	23	85 %	Ossur Low Profile Vari-Flex
S8	Trauma	33	65 %	Otto Bock Triton
S11	Vascular Disease	12	80 %	Ossur Flex-Walk

Subjects participated in three sessions: An initial fitting of the VI Knee, a practice walking session and the biomechanics testing session. Each session was performed on different days to avoid fatigue. All sessions were performed at the PVAMC Gait Lab.

In the initial fitting session, the VI Knee was attached to subjects’ existing socket and foot. All subjects used similar low-profile, carbon fiber energy storage and return feet (Table 1), which is not considered to cause significant differences in gait between subjects [30]. The prosthesis was attached and aligned by a certified prosthetist. The knee was then adjusted to the subject’s gait by tuning the initial swing acceleration, maximum swing velocity and terminal swing deceleration to produce an optimal gait that was comfortable for the subject and natural in appearance as determined by the prosthetist. Behavior of the VI Knee in stance was fixed across subjects and allowed for stance flexion of the device. These tuning values were recorded and used for the later practice and experimental sessions. Tuning was performed at each subject’s customary walking speed (1.13 ± 0.15 m/s across subjects). The fitting session lasted about an hour, at the end of which, subjects were able to assuredly walk with the VI Knee at variable speeds over clear, level ground. At this session, each subject’s height and weight was measured. The length and weight of their current prosthesis was also measured and its center of mass determined using the knife-edge balance technique by finding the balance point of the prosthesis with shank and the prosthetic foot [31]. This information was necessary for later use in the biomechanical model of each subject.

The practice sessions involved continuous walking at customary walking speed as well as the speeds to be tested - 1 and 1.25 m/s. The speeds tested were selected as they were fixed speeds slightly outside the subjects’ customary walking speed but were not overly laborious due to being too slow or too fast. Subjects started with a 30 min “free walk” where they were asked to walk at their customary walking speed in a large, cleared hallway around the building (approximately 120 m/lap, 1.25 m corner radius) to become further acquainted with the device. Rest periods were available as needed during the “free walk” portion of the experiment. After a 5 min rest, they then walked with the new device for 7 min at 1 and 1.25 m/s (randomly determined) with a 5 min rest between practice trials. This was repeated twice for a total experiment duration of about 2 h. The fixed walking speeds were controlled by having subjects keep pace with a member of the research team who used a metronome (via earphones so only they could hear it) calibrated to their gait such that they maintained a fixed speed with respect to their paced cadence. Subjects were instructed to keep pace with the pace-setter and to maintain a forward gaze so that they did not visually entrain to the pace-setter’s cadence.

In the testing session, 64 IR reflective markers were placed on the limb segments and joint centers of the subjects’ limbs and torso. The testing involved subjects walking along a 10 m walkway while their gait was recorded at 100 Hz using an infrared (IR) video motion capture system (Qualisys, Gotenberg, Sweden, Oqus 500 cameras). Force plates (AMTI, Watertown, MA) in the middle of the walkway recorded ground reaction forces and moments. Motion capture data was processed using Visual3D (C-Motion, Germantown, MD). Data processing of marker signals consisted of 10 sample (0.1 s) 3rd order polynomial spline-fit gap filling, applying a 6 Hz low-pass filter, and using automatic gait event detection [32]. Force plate data was used to compute gait kinetics by using the standard human body model provided by Visual3D, modified for each subject to reflect the differences in weight and location of center of mass of the prostheses investigated. Five of these walking trials with each prosthesis at both speeds tested were used to calculate mean values of parameters characterizing each subject’s performance.

Comparisons across subjects between the two knees tested were tested using a mixed model design ANOVA with the knees investigated as the fixed effects and subjects the random effects. Each subject’s individual performance difference with each knee was tested using Welch’s t-tests (to account for differences in variance between the conditions tested), with two tails and α = 0.05 were used to test significance between conditions (prosthesis). In all tests, p-values less than 0.05 were considered significant. Correlation comparisons were tested for significance as per [33]. Normality of data was checked using normal probability plots and was found to be normally distributed.

## Results

**Fig. 2:**
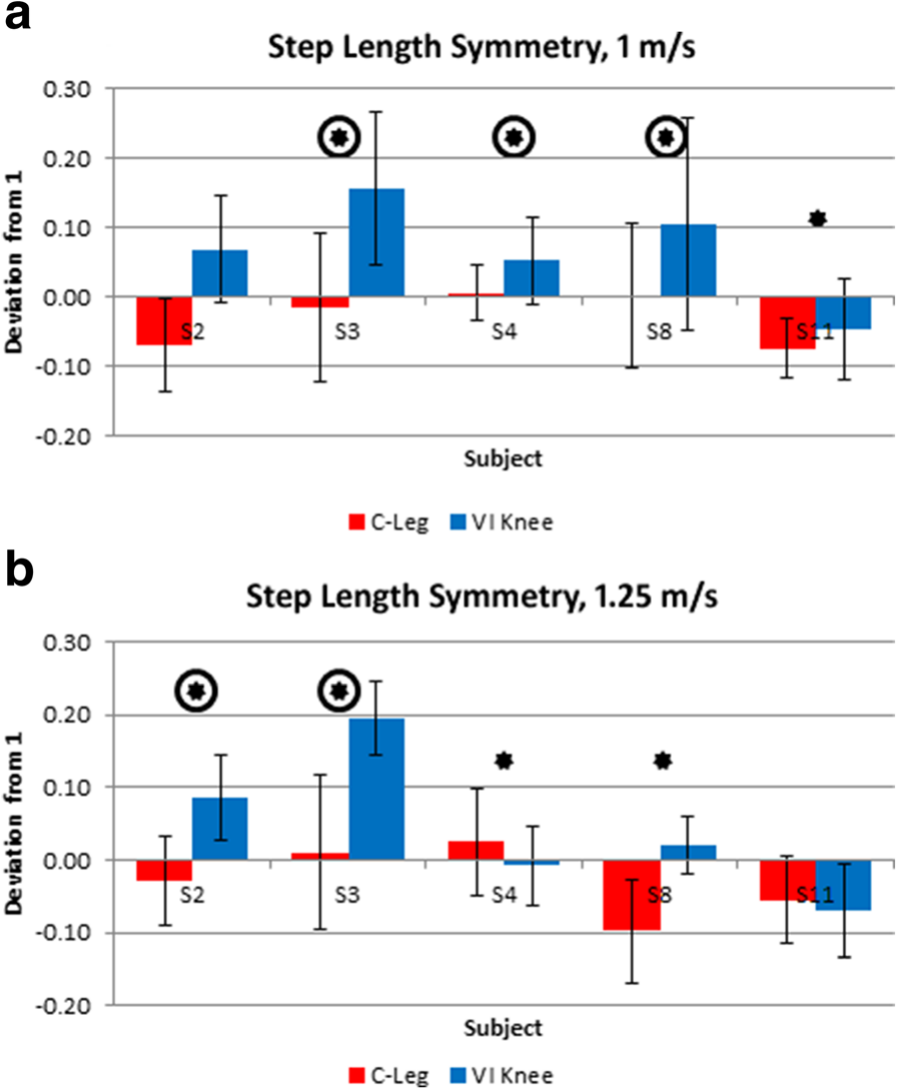
Deviation from 100 % step length symmetry between affected and intact sides for walking at 1 m/s (**a**) and 1.25 m/s (**b**). Significant differences (*p* ≤ 0.05) in symmetry magnitude are denoted by a star (improved symmetry due to using the VI Knee) or a circled star (reduced symmetry). Positive values indicate longer step lengths with the affected side leg (standing on the intact side) compared to the intact side, negative values denote shorter steps with the affected leg compared to the intact side

**Fig. 3:**
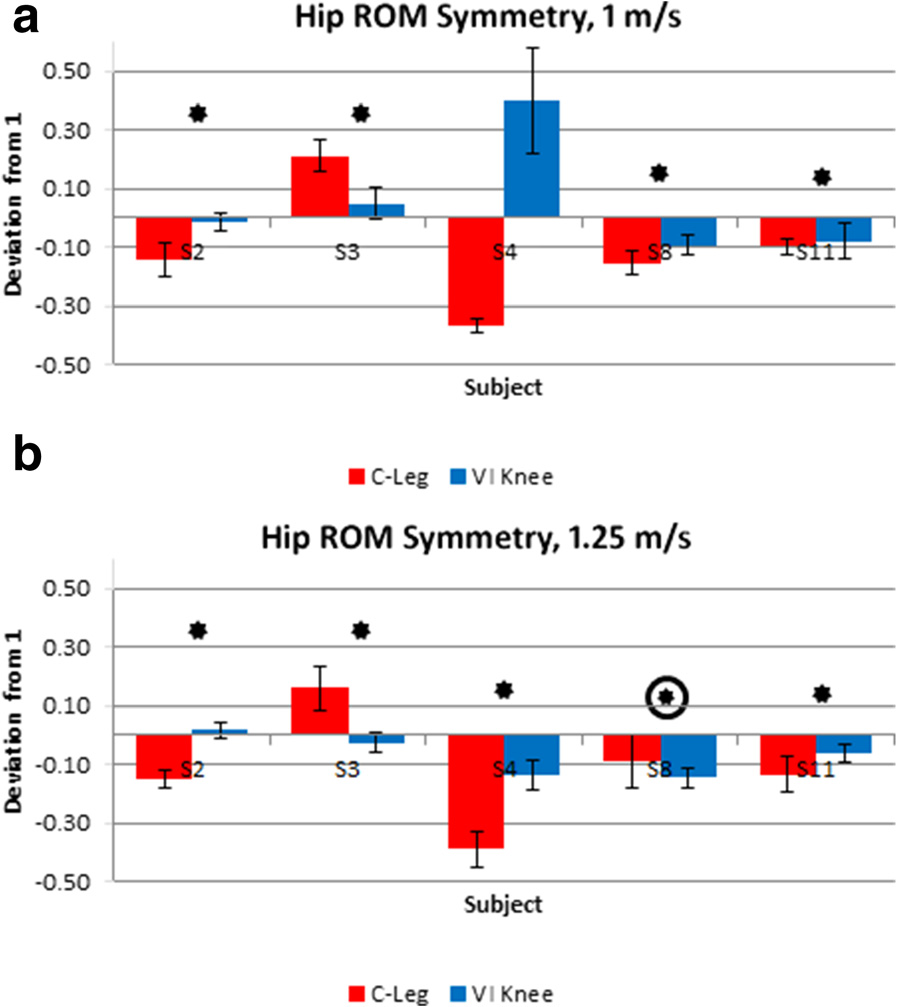
Deviation from 100 % hip range of motion (max flexion – max extension) symmetry between affected and intact sides for walking at 1 m/s (**a**) and 1.25 m/s (**b**). Significant differences (*p* ≤ 0.05) in symmetry magnitude are denoted by a star (improved symmetry due to using the VI Knee) or a circled star (reduced symmetry). Positive values indicate greater hip range of motion on the affected side, negative values, a smaller range of motion

**Fig. 4:**
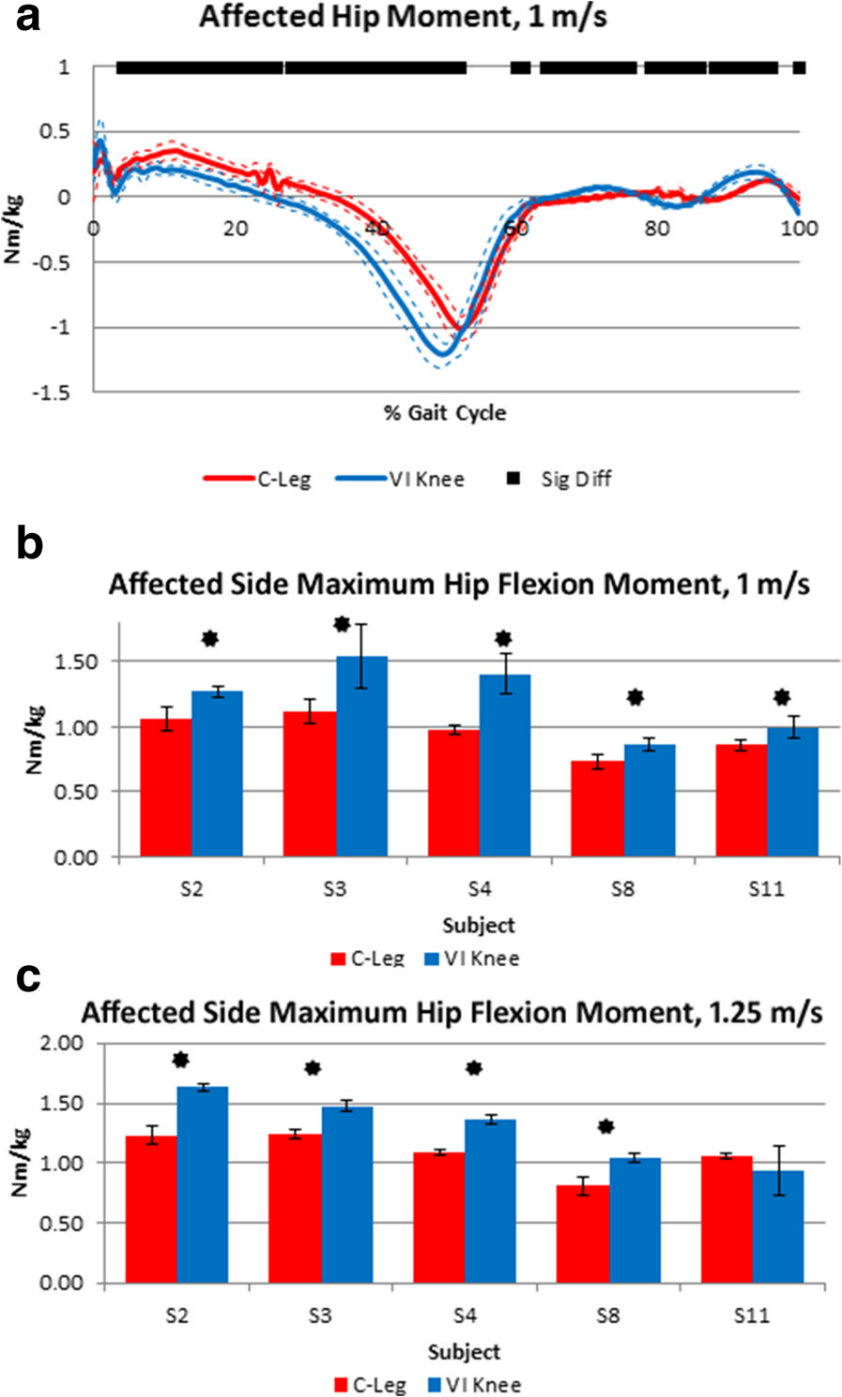
Representative plot of the affected side internal hip extension moment over the gait cycle for one subject at the slower walking speed (**a**). Solid and dotted lines represent the mean and standard deviation (±1 SD), respectively. Significant differences in the moments between the two knees tested are shown by the black points at the top of the plot. Affected side maximum terminal stance phase hip flexion moment, normalized to body weight, for walking at 1 m/s (**b**) and 1.25 m/s (**c**). Significant increases (*p* ≤ 0.05) in hip moment due to using the VI Knee compared to a C-Leg are denoted by stars

**Fig. 5:**
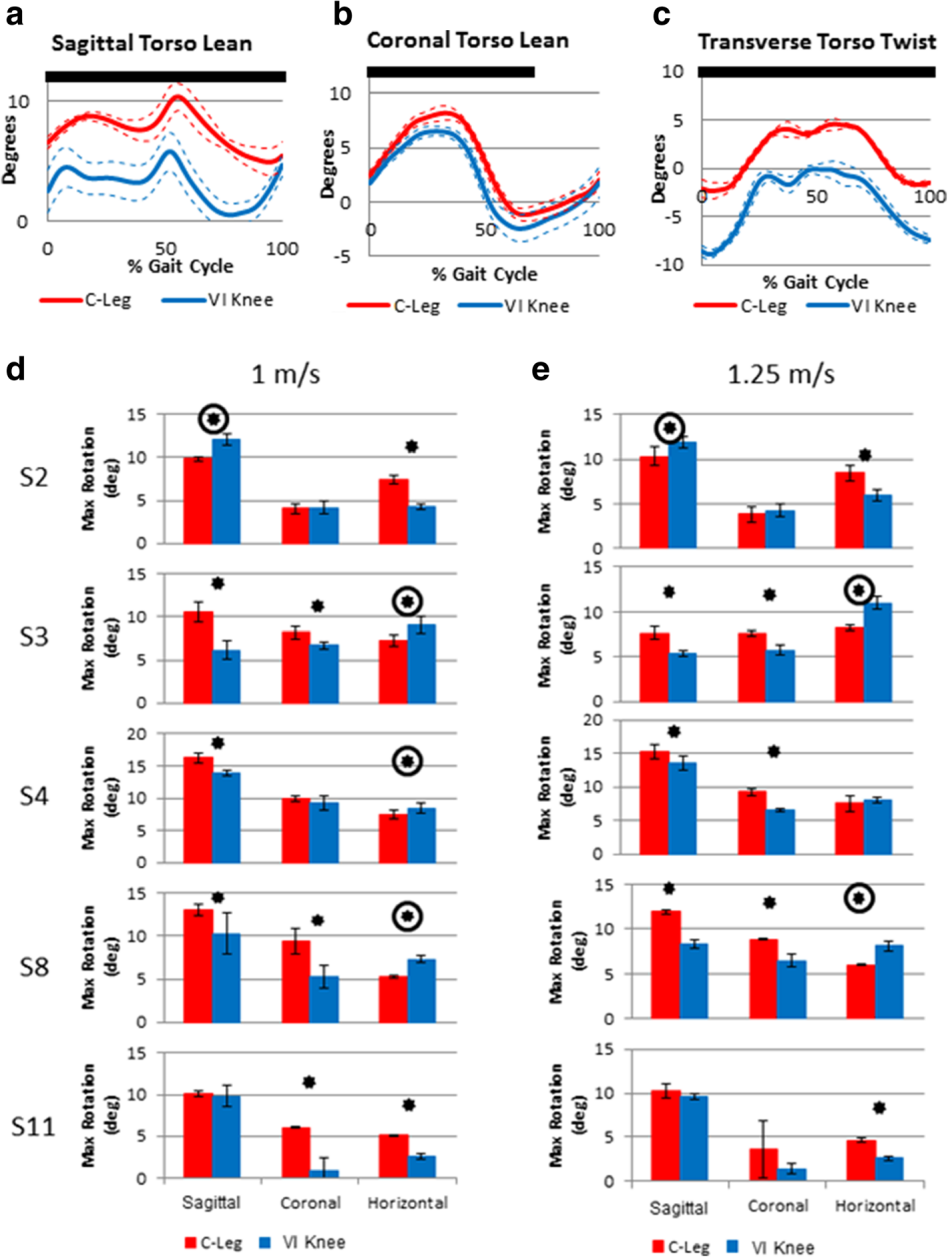
Typical torso lean and twist over the gait cycle for one subject (**a**-**c**). Solid and dotted lines represent the mean curve and standard deviation (±1 SD), respectively. In these plots, significant differences between conditions are indicated by a black bar at the top of the plot. Maximum upper body rotations about the pelvis during affected leg stance by subject for walking at 1 m/s (**d**) and 1.25 m/s (**e**). Positive sagittal and coronal rotations refer to lean forward and toward the affected side respectively. Transverse plane rotation of the torso about the pelvis rotating toward the stance leg. Significant (*p* ≤ 0.05) reductions (improved performance) in lean and twist are denoted by a star. Significant increases (worse performance) are noted by a circled star

**Fig. 6:**
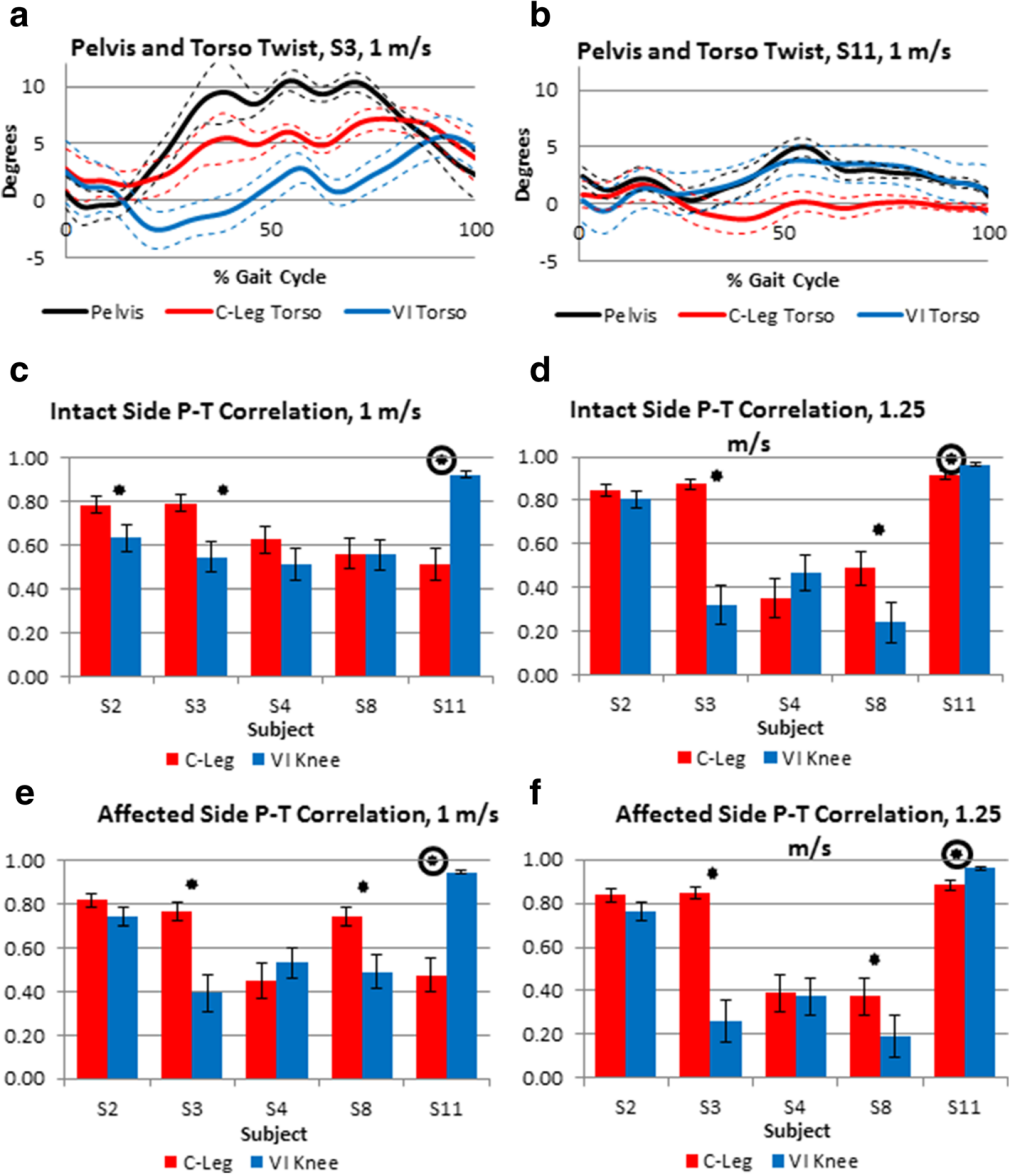
Plot of single subjects’ pelvis-torso (P-T) twist across the gait cycle for an individual who exhibited improved (decreased) pelvis-torso correlation due to using the VI Knee (**a**) and a participant who showed worsened (increased) P-T correlation while using the VI Knee (**b**) compared to the C-Leg. Ideally, the pelvis (black trace) and torso (blue and red traces) should be twisting in opposite directions over the gait cycle. Correlation for all subjects between pelvis and torso twist for steps taken with the intact side at 1 m/s (**c**), 1.25 m/s (**c**), and the affected side at 1 m/s (**d**) and 1.25 m/s (**e**). For sub-figures (**c**–**f**), significant (*p* ≤ 0.05) reductions (improved performance) in correlation are denoted by a star. Significant increases (worse performance) are noted by a circled star

Significant differences between prosthetic conditions were observed across all subjects (p values ranging from <0.0001 to 0.04) in the following: Step length symmetry between intact and affected sides (Fig. 2), hip range of motion symmetry between intact and affected sides (Fig. 3), affected leg hip extension moment (Fig. 4), prosthesis knee power (Table 2), upper body rotation (Fig. 5) and pelvis-torso twist correlation (Fig. 6). The significant observations of step length and hip ROM symmetry, trunk rotation and pelvis-torso twist correlations for each subject are summarized in Table 3. Each subject responded differently to the VI Knee, resulting in a large variability across subjects. However, statistically significant differences between the knees tested were observed. Throughout the text, values which represent improved performance while using the VI Knee compared to the C-Leg are denoted by a star (*). Similarly, those values that indicate worse performance while using the VI Knee compared to the C-Leg are indicated by a circled start (⊛).

**Table 2:** Maximum knee power during three sub-phases of gait normalized to body weight by prosthesis

Speed (m/s)		Pre-string power (W/kg)		Initial string flexion power (W//kg)		String extension power (W/kg)	
	Subject	C-Leg	VI Knee	C-Leg	VI Knee	C-Leg	VI Knee
1	S2	0.12 ± 0.02	0.16 ± 0.01 *	−0.58 ± 0.05	−0.41 ± 0.05 ⊛	0.01 ± 0.00	0.22 ± 0.10 *
	S3	0.09 ± 0.02	0.95 ± 0.27 *	−1.74 ± 0.29	−3.12 ± 0.43 *	0.20 ± 0.01	1.05 ± 0.08 *
	S4	0.15 ± 0.03	0.32 ± 0.16 *	−0.66 ± 0.11	−0.75 ± 0.10	0.14 ± 0.02	0.22 ± 0.03 *
	S8	0.09 ± 0.04	0.48 ± 0.07 *	−0.83 ± 0.09	−1.02 ± 0.09 *	0.25 ± 0.04	0.98 ± 0.07 *
	S11	0.08 ± 0.03	0.49 ± 0.23 *	−0.80 ± 0.09	−1.11 ± 0.20 *	0.19 ± 0.01	0.66 ± 0.06 *
1.25	S2	0.11 ± 0.02	0.13 ± 0.00 *	−0.88 ± 0.16	−0.69 ± 0.09 ⊛	0.01 ± 0.00	0.27 ± 0.02 *
	S3	0.13 ± 0.02	0.86 ± 0.17 *	−2.02 ± 0.11	−3.52 ± 0.12 *	0.20 ± 0.01	1.10 ± 0.07 *
	S4	0.21 ± 0.10	0.27 ± 0.03	−1.07 ± 0.26	−1.10 ± 0.08	0.23 ± 0.01	0.28 ± 0.01 *
	S8	0.20 ± 0.03	0.80 ± 0.12 *	−0.98 ± 0.08	−1.21 ± 0.04 *	0.38 ± 0.02	0.96 ± 0.09 *
	S11	0.09 ± 0.01	0.84 ± 0.39 *	−1.47 ± 0.03	−1.45 ± 0.27	0.18 ± 0.01	0.71 ± 0.01 *

Significant (p < 0.05) improvements are noted by an “*”, significant reductions are noted by a “⊛”

**Table 3:**
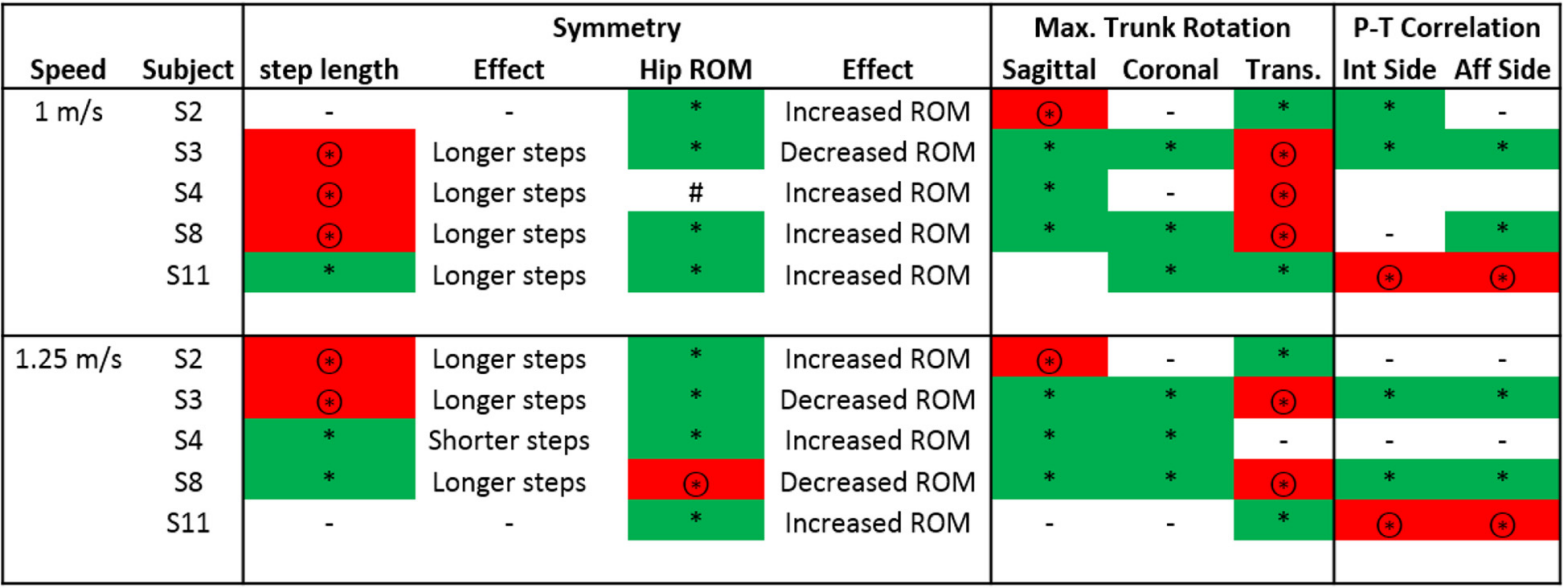
Summary of the significant effects on step length and hip range of motion (ROM) symmetry, maximum trunk rotation and pelvis-torso (P-T) rotation correlation due to using the VI Knee instead of the C-Leg

Worse conditions due to walking with the VI Knee are denoted with a ‘⊛’ and red shading, improvements due to using the VI knee are indicated by a ‘*’ and green shading. For Symmetry, more similar behavior between the affected and intact legs was seen as improved performance while less similar behavior a detriment. Improvements in Maximum Trunk Rotation and P-T Correlation are defined as reductions while using the VI Knee compared to the C-Leg. For subject S4 walking at 1 m/s, there was no significant difference in hip ROM symmetry (#), however, the individual did show a substantial increase in hip range of motion

Step length is defined as the forward distance of the heel at footfall from the preceding contra-lateral heel at its footfall (Fig. 1c). Step length symmetry is defined as the ratio of the affected side step length to that of the intact side. To better illustrate the differences, the deviation in symmetry (difference from 100 %) is presented. More positive values indicate a longer affected side step length, while more negative deviations from symmetry indicate a shorter affected side step. Ideally, the magnitude of the deviation from 100 % is as small as possible. At 1 m/s, one subject (S11) showed improvement in symmetry by taking longer steps with the VI Knee than with the C-Leg (Fig. 2a, starred). Three subjects showed decreased step length symmetry (S3, 4 and 8) while using the VI Knee at this speed, the result of taking longer steps with the affected leg (Fig. 2a, circled star). One subject (S2), showed no overall change in symmetry though they did exhibit a shift from shorter affected side steps with the C-Leg to longer steps with the VI Knee. Four of five subjects showed significant differences in step length symmetry at 1.25 m/s. Two subjects exhibited improved symmetry (Fig. 2b, starred), one by taking shorter steps with the VI Knee (S4) and one by taking longer ones (S8). Two subjects showed decreased step length symmetry (Fig. 2b, circled star) by increasing their step length with the VI Knee (S2 and 3) and one subject (S11) showed no significant difference between devices at the faster speed.

Hip range of motion (ROM) symmetry is defined as the ratio of the differences (maximum flexion – maximum extension) between affected and intact sides. Similar to step length, more positive values indicate a larger affected side hip range of motion, more negative values, a smaller range of motion. Again, closer to zero deviation from 1 is ideal. At 1 m/s, four of five subjects (S2, 3, 8 and 11) showed an improvement in hip ROM symmetry (Fig. 3a, starred). The subject who showed no statistical difference in symmetry (S4) did show a significant increase in ROM (but not symmetry) while using the VI Knee. At 1.25 m/s, four of five subjects (S2, 3, 4 and 11) exhibited improved gait symmetry (Fig. 3b, starred), while one had reduced symmetry (S8) while using the VI Knee at this speed (Fig. 3b, circled star).

Hip flexion moment during terminal stance is associated with sagittal plane (forward) torso lean, with less lean producing a larger flexion moment [34]. While greater moment is required by the hip, maintaining a more vertical posture is more beneficial to overall comfort. At both speeds tested, subjects exhibited increased affected side hip flexion moment in late stance while using the VI Knee (Fig. 4). Subjects typically exhibited both decreased hip extension shortly after heel strike and greater maximum flexion moment in terminal stance (Fig. 4). At 1 m/s, all five subjects showed significant increases in maximum hip flexion moment (Fig. 4b, starred). At the faster 1.25 m/s, four of five subjects (S2, 3, 4 and 8) showed significantly increased maximum hip flexion moment (Fig. 4c, starred). Subject S11 showed no significant difference in hip flexion moment between conditions at this speed.

The robotic VI Knee showed increased knee power generation across most subjects and both speeds during pre-swing, and swing extension and absorbed more energy during initial flexion compared to the passive C-Leg (Table 2). At 1 m/s, the VI Knee generated significantly greater power during pre-swing and swing extension (starred values). Maximum power absorbed during initial swing at this speed was significantly greater for three subjects (S3, 8 and 11, starred values) and less for one subject (S2, denoted on Table 2 with a ‘⊛’). Walking at 1.25 m/s, the knee generated significantly more power during pre-swing for four of five subjects (S2, 3, 8 and 11) and all subjects during swing extension. During initial swing flexion, two subjects had significantly greater power absorption (S3, and 8) at this speed and one subject had less (S2).

Across speeds, subjects showed differences in the maximum torso lean angle in both the sagittal (forward) and coronal (lean toward the affected side) planes as well as torso twist relative to the pelvis while using the VI Knee. Typical torso lean and twist angles as a function of the gait cycle for one subject is shown in Fig. 5a–c. Across all subjects, at both 1 and 1.25 m/s, three of five subject exhibited reduced forward lean while using the VI Knee (S3, 4 and 8, Fig. 5d & d, starred). One subject significantly increased their forward lean (S2, Fig. 5d and e, circled star) and one subject (S11) showed no difference.

At 1 m/s, three subjects had a reduced lean to the affected side while using the VI Knee (S3. 8, and 11, Fig. 5d, starred). Two subjects showed no significant difference due to using the powered device at this speed (S2 and 4). At the faster speed, again three subjects showed significantly reduced affected side lean (S3, 4 and 8, Fig. 5e, starred) and two subjects exhibited no significant difference (S2 and 11). No subjects exhibited increased coronal plane lean while using the VI Knee at either speed tested.

At both speeds, two subjects exhibited a reduction in maximum torso twist (transverse plane rotation) while walking with the VI Knee (S2 and 11, Fig. 5d and e, starred). At the slower walking speed, three subjects generated a larger maximum torso twist when using the robotic device (S3, 4 and 8, Fig. 5d, right column, circled star). Similarly, walking with the VI Knee caused an increase in maximum torso twist in two subjects at the faster walking speed (S3 and 8, Fig. 5e, circled star) and no significant difference in one subject (S4).

Figure 6a and b shows the transverse rotation angles (twist) between the pelvis and torso for single subjects over the gait cycle while using each knee prosthesis. In Fig. 6a, the subject demonstrates decreased correlation between the pelvis and torso twist. The subject in Fig. 6b illustrates increased correlation in the direction of twist of the pelvis and torso while using the VI Knee. The Pearson’s correlation coefficient between the torso and pelvis twist for all subjects over each step (heel strike to heel strike) is shown in Fig. 6c–f. In these graphs, values closer to 1 represent tighter coupling between pelvis and torso twist and that they are rotating together (in phase), whereas values closer to zero represent looser coupling,, and negative values approaching −1 (anti-correlated) indicate the pelvis and torso are twisting in opposite directions (out of phase). For reference, able-bodied individuals tend to have a pelvis-torso correlation of about −0.7 and are thus anti-correlated during normal gait with the pelvis and torso generally rotating out of phase in opposite directions [7]. At 1 m/s, for steps taken with the intact leg, two subjects show a significant reduction in pelvis-torso correlation (S2 and 3, Fig. 6c, starred) while one subject exhibited a significant increase in pelvis-torso correlation (S11, Fig. 6c, circled star). Two subjects (S4 and 8) showed no significant difference in intact side pelvis-torso correlation at this speed. Similarly, at 1.25 m/s two subjects (S3 and 8) had reduced correlation and one (S11) increased (Fig. 6d) and two subjects (S2 and 4) showing no significant differences between devices. For steps taken with the affected side, at both 1 and 1.25 m/s, two subjects (S3 and 8) showed significantly reduced pelvis-torso correlation (Fig. 6e and f, starred) and one subject (S11) significantly increased their pelvis-torso coupling (Fig. 6e and f, circled star). Two subjects (S2 and 4) displayed no significant difference in affected side pelvis-torso correlation across both speeds.

## Discussion

This study investigated the biomechanical differences between using the variable impedance VI Knee compared to the current standard of care for active walkers (OttoBock C-Leg). Significant differences were observed between conditions, with most of the five subjects tested showing improvements due to using the VI Knee in increased hip range of motion symmetry, increased prosthesis knee power and reduced sagittal and coronal plane lean as well as increased terminal stance hip flexion moment (an indicator of more upright gait posture). Mixed results were observed regarding the impact of using the VI Knee on pelvis-torso twist correlation, with two of five subjects showing improvement and one a worsening of performance. Significant disadvantages to using the VI Knee, where most subjects exhibited worse performance were observed in decreased step length symmetry and increased torso twist at the slower speed.

Individuals with an above knee amputation tend to exhibit a greater amount of torso rotation (in all three planes) and decreased step-length symmetry (by taking shorter steps on the affected side) compared to able-bodied subjects [7, 35] in part to compensate for weak hip abductors on their affected side due to disuse atrophy. By leaning (primarily toward the affected side), they are able to use the upper body to help lift the intact side hip to assist in clearing the toe. This population also shows a significant increase in in-phase coupling of the pelvis and torso compared to able-bodied individuals, due to an overall stiffening of the entire upper body during gait [7, 35]. Reasons for this stiffening range from a psychological/guarding behavior to the use of the upper body to control the motion of the pelvis and leg due to hip weakness [7]. This coupling of body segment rotation can have negative impacts on gait by increasing mechanical energy required for walking [36] and potentially reduces customary walking speed [7]. While using the VI Knee, the majority of subjects exhibited improved hip range of motion symmetry between intact and affected sides, (Fig. 3), reduced forward and affected side torso lean (Fig. 5). Mixed results were seen in the amount of coupling between the torso and pelvis twist with two of five subjects showing improvement and one worse performance (Fig. 6). The ability of the VI Knee to produce greater power compared to passive knees (Table 2) is potentially one difference between it and passive knees like the C-Leg that enables these improvements and allows for a more natural gait, unburdens the upper body from the gait task, and helps to lift the lower leg to clear the toe. As the biomechanical changes often associated with prosthetic use can lead to chronic hip and back pain and osteoarthritis of the hip and intact-side knee [8, 9], it is possible that improvements in hip ROM symmetry, with long term use of the VI Knee compared to a standard of care, passive knee prosthesis, may lead to reductions in the likelihood of these conditions arising. While some subjects exhibited increased torso twist, which has been linked to back pain in individuals with an above knee amputation [35], none of the subjects increased their torso twist to a magnitude or by an amount shown to cause back pain (15.4° ± 4.2° vs. maximum 10.85° ±0.68° observed in this study).

Across both speeds, most subjects increased their affected side step length when using the VI Knee (Fig. 2, Table 3), however in some cases this resulted in a decrease in symmetry. At the slower speed, most of those who took longer steps with the affected side leg were also the ones who showed an increase in maximum torso twist (Table 3). At the faster speed, this is also shown by two subjects (though one showed an improvement in symmetry by taking shorter affected side steps). In these subjects, increased rotation of the pelvis (relative to the torso) serves to increase step length. At both speeds, of the five instances of reduced step-length symmetry, four also occurred with increased torso twist (the fifth occurring with a reduction in maximum twist, but an increase in hip range of motion, Table 3). Further training may allow them to reduce their torso twist and thus improve step length symmetry.

Across speeds, most to all subjects increased their affected side maximum stance phase hip flexion moment (Fig. 4). This is consistent with the reduced forward torso lean observed in most subjects (Fig. 5). While not a direct assessor of overall gait performance itself, it is a result of walking with a more upright posture [34]. Increased terminal stance hip flexion moment is a result of needing to balance the ground reaction force which shifts posteriorly with less forward torso lean such that the line of action is more behind the hip. This allows the walker to maintain the center of pressure under the forefoot prior to weight transfer to the other foot. Comparing Figs. 4 and 6, those subjects who showed more reduced forward torso lean while using the VI Knee also exhibited more increased terminal stance hip flexion moment.

Based on the data collected, the VI Knee demonstrates potential advantages in knee power over a passive device (Table 2) including the generation of net positive power during pre-swing to help raise the center of mass. The device also absorbed more power than a passive device during initial swing flexion and generated positive power during swing, such that while being heavier than the conventional device, subjects tended to feel that the VI Knee was more responsive and had “less weight” than their C-Leg.

It should be noted that while significant differences were observed in the biomechanics of gait while using the two devices compared in this study, limitations do exist. Given the relatively small number of subjects studied and the initial findings from this work, a future study using the described methods with a larger population of PWAs above the knee, as well as age-height-weight matched able-bodied controls would be beneficial to more fully explore the biomechanical effects of using the VI Knee. While a variety of subjects with different residual limb lengths participated in this study, additional subjects would also allow for better observation of how limb length impacts VI Knee walking performance. Another particular issue with the current work is the amount of practice subjects received with the VI Knee. While all subjects walked with the VI Knee for several hours to become acquainted with the device, a longer practice time lasting days to weeks, including at home use, with testing before, during and at the end of this period would be a good next step for future work. It’s possible, that with a longer habituation time, users could become more comfortable with the VI Knee and better take advantage of its capabilities with the end result of reducing or eliminating the detriments to performance observed and further improving walking performance over the C-Leg. Another application to investigate with the VI Knee would be its combined use with a powered foot-ankle prosthesis. Such a combined system, with synchronized operation between the two devices could potentially allow for more complete replication of natural gait further enhancing the benefits observed in this work.

## Conclusion

PWAs above the knee tend to walk with marked gait asymmetry and with increased upper body involvement compared to able-bodied individuals. Over time, these gait changes can lead to chronic hip and lower back pain and osteoarthritis. While current prostheses allow users to walk with improved gait over older devices and recently developed powered devices have shown increased knee power, they have not been able to fully restore typical gait to PWAs above the knee. Towards this end, the preliminary data on five subjects from this study demonstrate that the VI Knee may have the potential to improve gait symmetry and reduce upper body involvement in PWAs above the knee compared to current standard of care devices such as the C-Leg. While using the VI Knee, most subjects demonstrated significant improvement in reducing upper body involvement in the walking task by decreasing forward and affected side lean, and by increasing hip range of motion symmetry between affected and intact sides. Subjects also exhibited detriments to correct gait including decreased step length symmetry. These effects may be mitigated through longer term training and experience with the device. Based on the observed benefits shown in this preliminary work and the potential to reduce or eliminate detrimental effects through training, the data suggest that using a powered device like the VI Knee, particularly over an extended period of time, may help to improve walking comfort and reduce the likelihood of chronic hip and back pain and osteoarthritis due to using a knee prosthesis.

## Abbreviations

IR, infrared; PWA, person with amputation; ROM, range of motion; VI Knee, variable impedance knee
